# Children's use of gesture in ambiguous pronoun interpretation*

**DOI:** 10.1017/S0305000915000045

**Published:** 2015-02-20

**Authors:** WHITNEY GOODRICH SMITH, CARLA L. HUDSON KAM

**Affiliations:** University of Maryland, College Park; University of British Columbia

## Abstract

This study explores whether children can use gesture to inform their interpretation of ambiguous pronouns. Specifically, we ask whether four- to eight-year-old English-speaking children are sensitive to information contained in co-referential localizing gestures in video narrations. The data show that the older (7–8 years of age) but not younger (4–5 years) children integrate co-referential gestures into their interpretation of pronouns. This is the same age at which they show sensitivity to order-of-mention, the only other cue available in the stimuli. Interestingly, when children show sensitivity to the gestures, they are quite similar to adults, in that gestures consistent with order-of-mention increase first-mentioned responses as compared to stimuli with no gestures, but only slightly, while gestures inconsistent with order-of-mention have a larger effect on interpretation, decreasing first-mentioned responses and increasing second-mentioned responses.

## INTRODUCTION

A wide body of research has demonstrated that co-speech gestures, the natural and spontaneous hand movements that co-occur with speech, are more than just random hand waving (e.g. McNeill, 1992, 2005); gestures contain information related to that expressed in the verbal modality. Sometimes gestures convey information that is congruent with that present in the speech, but not always. There is ample evidence that listeners are able to integrate information presented in gesture with that presented in speech to form a unified interpretation, even when the two modalities contain different information (Cassell, McNeill & McCullough, 1999; Goodrich Smith & Hudson Kam, 2012; Singer & Goldin-Meadow, 2005). This integration can lead to improved comprehension (Beattie & Shovelton, 1999; Campana, Silverman, Tanenhaus, Bennetto & Packard, 2005), especially in cases where the gestures help to clarify ambiguity (Goodrich Smith & Hudson Kam, 2012; Thompson & Massaro, 1994).

Previous work has shown that the ability to integrate information from the two modalities is not restricted to mature language users. Morford and Goldin-Meadow (1992), for example, found that even children at the 1-word stage can integrate information from speech and a pointing gesture; when they said *push* to children while pointing toward a ball, the children responded by pushing the ball. The current study expands on the previous findings by asking whether, like adults, young children can also use information present in more abstract gestures to help resolve ambiguities in the speech. We examine this with respect to abstract ambiguities, namely ambiguities associated with pronominal reference. Research shows that speakers may gesture in a location in space when referring to an entity and then gesture back to the same location when referring to the same entity again later in the same discourse (Kendon, 2004; McNeill, 1992; So, Kita & Goldin-Meadow, 2009), something we have called co-referential localizing gestures (Goodrich Smith & Hudson Kam, 2012). These gestures are particularly interesting to us because they provide potential cues to the intended referent of anaphoric pronouns. Pronominal reference is an aspect of language that children normally have some difficulty with (e.g. Hendriks & Spenader, 2005/2006; Karmiloff-Smith, 1986), and it is possible that the information provided by co-speech gestures might be particularly useful, helping the child figure out the speaker's intensions, and thus resolving the referential ambiguity.

### Background

Pronouns like *he* and *she*, common in everyday speech, do not consistently map onto any referent. Their meaning is instead determined anew each time they are used. Although pronouns are often quicker and easier to pronounce than full noun phases, making speaking more efficient, they can also introduce a degree of ambiguity to our language, potentially making comprehension more difficult. This is particularly true when a speaker uses a pronoun after describing more than one person. In (1), for example, it isn't clear whether the visit occurred during John's or Sam's summer vacation.

(1) John visited Sam during his summer vacation.

However, most of the time adult listeners do not appear to notice ambiguities and are quickly and easily able to infer the speaker's intended meaning. Researchers have described a variety of cues present in the speech that affect pronoun interpretation. Some of the cues that have received a great deal of attention in the literature are gender (i.e. knowing that *she* refers to a female antecedent; Arnold, Eisenband, Brown-Schmidt & Trueswell, 2000; Crawley, Stevenson & Kleinman, 1990; Ehrlich, 1980), emphatic stress (for instance, in the sentence *John hit Harry, and then Sarah hit him*, most adults interpret the referent of the pronoun to be Harry, unless *him* is stressed, in which case John becomes the preferred referent; Maratsos, 1973), grammatical function (e.g. pronouns often refer to the grammatical subject of an utterance; Gordon, Grosz & Gilliom, 1993), and order-of-mention (the tendency for a pronoun to refer to the first-mentioned entity, which is frequently also the grammatical subject; Arnold *et al.*, 2000). While the cues just mentioned are part of the structure of the sentence, others are more pragmatic or semantic in nature. For instance, listeners prefer to interpret pronouns as being co-referential with the topic, even in a language like English which lacks specific topic-marking syntax or morphology. And the nature of the event being described by the verb, in conjunction with aspect in the sentence, can also affect interpretation preferences, even to the point of overriding grammatical function and order-of-mention (Ferretti, Rohde, Kehler & Crutchley, 2009; Rohde, Kehler & Elman, 2006). It is quite clear from the literature on pronoun resolution that it is not a simple process, and that a wide range of information can affect pronoun interpretation.

Recent theories such as Arnold's (1998, 2001, 2010) Expectancy Hypothesis highlight the dynamic and often interactive influence of cues on the listener's understanding of the speaker's intensions and how those expectations affect interpretation. On this view, cues make certain antecedents more accessible, linguistically prominent, or salient, or provide listeners with hints as to what the speaker is focusing on. This view has the ability to accommodate the impact of structural cues such as grammatical role, and pragmatic/semantic cues like event structure, as well as the fact that different information seems to be more or less important in different contexts (see, e.g. Pyykkönen, Matthews & Järvikivi, 2010).

Although gesture does not fit neatly into notions of accessibility, linguistic prominence, or salience, it does provide information about the speaker's mental model underlying the speech, which might be useful to the listener in constructing her own mental model of the discourse. This kind of information is also accommodated by the Expectancy Hypothesis; anything that is relevant and can be interpreted by a listener has the potential to affect interpretation. Indeed, there are data from adults showing that other things which do not fit neatly into accessibility hierarchies or cue-based prominence but which do involve listeners making inferences about the speaker's mental model (namely disfluency) affect interpretation processes, demonstrating the validity of considering things outside the structure and semantics of the speech itself for understanding natural language understanding (Arnold, Hudson Kam & Tanenhaus, 2007).

Goodrich Smith and Hudson Kam (2012) found that adult listeners' interpretation of ambiguous pronouns is affected by the presence of co-referential localizing gestures, a cue that is completely outside the speech signal, but one which carries information about the speaker's intended message. In that study, adult listeners watched a narrator telling a story involving multiple characters of the same gender. The narrator produced gestures in distinct locations in space (on the Left–Right axis) when introducing each character by name. She then gestured back towards one of the locations when describing an activity performed by a single character who was referred to using a pronoun. Despite a clear bias to interpret the pronouns as referring to the first-mentioned character in the absence of any gestures as well as when the gesture indicated the first-mentioned character was the intended referent (68% of the time with no gestures, 72% of the time with the gestures), when listeners saw a gesture that went against order-of-mention, they often interpreted the pronoun as referring to the second-mentioned character, doing so 38% of the time, which was more than twice as often as participants in the other two conditions, who did so only 14% of the time.

Developmental studies show that children do not initially use speech-internal cues in the same way as adults. Indeed, it takes time for children to learn the cues that adults so readily use to interpret ambiguous pronouns. Stress, for instance, does not seem to guide interpretation in adult-like ways until late childhood. Maratsos (1973) had three-year-old children act out sentences such as *John hit Harry, and then Sarah hit him*. While the children were able to successfully act out sentences with unstressed pronouns, they did not correctly interpret stressed pronouns: even when *him* was stressed, they interpreted it to mean Harry. In a similar study, Kertoy (1991) compared older children's comprehension of contrastive stress versus topic continuity. She found that first-grade children were at chance in interpreting co-referential pronouns, and that they did not appear to rely on prosodic information at all. By fifth grade, children were interpreting the normally stressed pronouns co-referentially 86% of the time (a rate close to adults), but when the pronoun was stressed, they did not shift their interpretation away from the previously introduced topic; that is, they continued to interpret *him* as Harry.

The results for order-of-mention are somewhat conflicting. Arnold, Brown-Schmidt, and Trueswell (2007) found that four- and five-year-old children (and girls, but not boys, aged 3;6–4;0) correctly interpreted pronouns when they were marked by gender, but, unlike adults, failed to show a preference for one character versus the other when the pronoun matched the gender of both. The children showed the highest level of performance in situations where both gender and order-of-mention pointed to the same referent, an idea supported by the work of Song and Fisher (2005, 2007). In a looking-time experiment, Song and Fisher (2005) found that three-year-old children tended to prefer the grammatical subject from the preceding context as the referent for an ambiguous pronoun, especially when the grammatical subject was also the first-mentioned character. This suggests that while order-of-mention may not be fully acquired by age three, children at that age do have some sensitivity to the cue. But in a follow-up pilot study with the same stimuli, Song and Fisher (2007) found that children aged 2;6 actually showed a slight bias to prefer the most recently mentioned referent, rather than the grammatical subject/first-mentioned character. However, when the stimuli were revised, even children aged 2;6 were successful at identifying the correct referent for a pronoun when it was singled out as being more prominent than the other referent through a variety of co-occurring cues (e.g. by having a higher frequency of mention and/or being mentioned first, and in the subject position). Thus, children seem to perform best when several cues point to the same interpretation.

Research by Pyykkönen *et al.* (2010) is also relevant here. They investigated whether three-year-old English-speaking children's pronoun interpretation is sensitive to the semantic prominence of the various possible antecedents, in addition to grammatical role/order-of-mention, also using an eye-tracking task. They manipulated semantic prominence by varying the nature of the action encoded by the (transitive) verb in the sentence in which the possible antecedents were introduced. Some actions made both subject and object semantically salient (high-transitivity verbs), while others made neither particularly salient (low-transitivity verbs). They found that when antecedents were introduced in a sentence with a high-transitivity verb, the subject and object (or first- and second-mentioned) nouns were equally likely to be looked at early on in the test sentence containing the pronoun, and were looked at more than if they had been introduced using a low-transitivity verb. However, later on in the sentence (i.e. more time after the hearing the pronoun), the subject/first mentioned noun was fixated on more than the object/second-mentioned noun, and this split happened more quickly in the low-transitivity than the high-transitivity condition.

Interestingly, order-of-mention appears to be playing a role at a younger age in the Pyykkönen *et al.* (2010) and Song and Fisher (2005, 2007) studies than in the Arnold *et al.* (2007a) study. Although it is not entirely clear why the various studies find sensitivity to the speech-internal cues at such different ages, Hartshorne, Nappa, and Snedeker (2011) point out that Arnold *et al.* (2007a) used a relatively short time window for interpretation compared to the Song and Fisher studies, which is consistent with the Pyykkönen *et al.,* data. Thus, it would seem that order-of-mention is a relatively weak, but available, cue in younger children (see, e.g. Beyer & Hudson Kam, 2009; MacWhinney, Bates & Kliegl, 1984).

In many respects, young children's relative failure to use speech-internal (or structural) cues is unsurprising. Such cues are only probabilistically related to interpretation, and their correlation with anaphoric relationships must be learned (Arnold *et al.*, 2007a): pronouns do not always refer to grammatical subjects or first-mentioned entities; stress on the pronoun indicates only that the intended referent is different from the usual pattern, it does not directly point to the referent; even gender only serves to limit the set of possible referents. Gesture, on the other hand, is more concrete in that, at least metaphorically, it points directly to the referent, and although not always available, will likely generally indicate the intended referent accurately. That is, it is a more direct link with the speaker's intended pronoun reference than the order in which they place referents in a prior sentence. On these facts, gesture might be expected to influence pronoun interpretation in children quite early.

Moreover, there is ample evidence from a wide range of studies that children are sensitive to gestures very early on. By 12 months, infants are not only producing pointing gestures themselves, but also understand the points of others (e.g. Behne, Liszkowski, Carpenter & Tomasello, 2012). By 1;6, children understand gesture and speech combinations, where a pointing gesture adds new information to speech, such as ‘push’ along with a deictic gesture to a ball (Morford & Goldin-Meadow, 1992). As children grow older, they also become sensitive to more abstract gestures. For instance, two-year-old children can rely on an iconic gesture to infer the meaning of a new verb (Goodrich & Hudson Kam, 2009), and by age three, children begin to comprehend iconic gesture plus speech combinations, such as “I am eating” while a closed fist scoops toward the mouth to represent cereal (Stanfield, Williamson & Özçalişkan, 2014). Thus, we might expect this highly informative cue regarding a speaker's communicative intentions to be useful for children in interpreting pronouns, even at a young age.

## THE CURRENT STUDY

In the current study we examine whether gesture influences pronoun interpretation in children, especially at an age when they are still acquiring the speech-internal cues. In particular, we examine whether children aged four to eight can use co-referential localizing gestures to interpret pronouns. We investigated this by having the children watch videos in which a narrator told a story involving two characters. The characters were introduced using names or other specific forms, mentioned again by name in a subsequent sentence, and at some point later in the story one was referred to using a pronoun. We assessed participants' interpretation of the pronoun by asking who performed the action described in the sentence containing the pronoun. In two conditions (described shortly) the narrator gestured while introducing the characters and saying the pronoun, in a third condition she did not. We took care to minimize the potential impact of other cues. The referent of the pronoun was never disambiguated through context, and there was no clear topic to rely on. Both characters were of the same gender, and both were introduced in a similar grammatical and semantic role (e.g. both as subjects and actors). And as we were interested in typical interpretation, pronouns were never stressed.

One cue that can never be removed from a discourse, however, is order-of-mention; one character will always have to be mentioned first. This has some implications for our study. First, it means that our control or baseline condition, the no-gesture condition, actually contains a potential cue. Although most studies examining order-of-mention have used stimuli in which the first-mentioned character was highlighted in some additional way, for example, by being the grammatical subject, suggesting that order-of-mention alone may not influence interpretation, Goodrich Smith and Hudson Kam (2012) demonstrated a pure first-mentioned bias in adults. Thus, although order-of-mention is not the explicit focus of our study, our control condition does allow us to explore the development of the order-of-mention tendency in isolation from other cues, and in a wider age range than has previously been studied using a single methodology. Second, it means that we may not actually examine the impact of gesture alone on interpretation. If children have not yet picked up on the first-mentioned pattern, then gesture alone may influence their interpretation. However, if they have even limited sensitivity to the first-mentioned pattern, as is suggested by the results of some previous studies, then we are examining how gesture interacts with order-of-mention.

Here we are explicitly assuming that a true first-mentioned bias (that is, when the only factor is order-of-mention) is learned via some sort of statistical learning. We know that speakers implicitly pick up on patterns in the language over time, and that these often abstract patterns can influence language processing and are manipulable (see, e.g. Reali & Christiansen, 2007; Wells, Christiansen, Race, Acheson & MacDonald, 2009). Moreover, implicitly learned patterns are not always communicatively relevant (see Ellis, 2002, for a discussion). If pronouns tend to refer to the first-mentioned characters (likely due to their being subjects), which data suggest that they do (Arnold, 1998), this pattern is available to be learned. That is, we see the pure order-of-mention condition as a side effect of other aspects of pronoun use, not a primary cue of pronoun use. As such, we would expect it to be a weak cue, or at least to produce a weak effect when it is the only cue, and one which is rather easily overridden by more communicatively relevant information, something which is true of the adult data reported in Goodrich Smith and Hudson Kam (2012).

Given the previous work on pronoun interpretation in children, as well as the work on speech gesture integration, it is not entirely clear what to expect in this study. On the one hand, we might expect the youngest children in our study to fail to use gesture to guide interpretation at all, since at the early ages included in our study pronouns may be topically rather than anaphorically interpreted (see Karmiloff-Smith, 1986), and so guides to anaphoric linkages might be ignored. The distinction is subtle but important. An anaphoric pronoun's reference is dependent on another referential element in constrained and semi-predictable ways. Children often seem instead to treat pronouns as referring to the referent itself, most often the topic of conversation, rather than referring to the referent through another referring expression (see Karmiloff-Smith, 1986). This can be seen in younger children's frequent use of a pronoun to introduce a referent, that is, using a pronoun without first naming or otherwise identifying a referent. If this is the case, then we might expect the youngest children to be unaffected by the gestures because they are inherently co-referential. Alternatively, younger children might fail to use gesture, not because of how they process pronouns, but rather because the types of gestures used in the study may be too complex for them to interpret. In order to successfully rely on the gesture to interpret a pronoun, children must be able to associate the space in which a gesture occurred with the character mentioned while the gesture was produced, and then later remember which spaces were associated with which characters. This is quite complicated, especially from a memory perspective, possibly too complicated for the youngest children. The gestures in our study are more abstract than points to actual physical objects and iconic representations of objects or actions, gesture forms that the youngest children in our study are known to interpret (e.g. Butterworth & Grover, 1988; Namy, Campbell & Tomasello, 2004). Evidence from the acquisition of signed languages shows that children can learn and so can interpret spatial co-reference of the sort investigated here, but it is not fully acquired until at least four years of age, even in children who are exposed to spatial co-reference consistently (Newport & Meier, 1985; Schick, 2003). Thus, for any of these reasons, we might expect to see the youngest children fail to incorporate the information from the gestures into their interpretation. If this is the case, and they also do not yet have the first-mentioned bias, then chance interpretation is expected, as there are no other cues available to guide interpretation. If they do already show a first-mentioned bias, in contrast, then we anticipate that they will prefer the first-mentioned character no matter the gesture.

On the other hand, gestural cues need not be learned like the speech-internal cues we've discussed – they are available to use to guide comprehension as long as they can be interpreted – and so they might be easily available to children as cues to pronoun interpretation even at a young age. Indeed, there is evidence to suggest that young children are capable of comprehending and remembering relatively complex gestures, and can utilize those gestures to interpret challenging aspects of language (Goodrich & Hudson Kam, 2009). Thus, it is also possible that the youngest children will interpret the pronouns according to the information contained in the gestures. If they do not yet have a first-mentioned bias, then interpretation will be at chance only when there are no gestures in the stimuli, otherwise they will interpret the pronouns according to the gestures. It is possible that the gestures might even play a stronger role in interpretation for the younger children as compared to the older children; if the first-mentioned bias is developing over this time period, and the younger children only have access to the gesture cue, it could more strongly influence interpretation. If, however, even the youngest children have sensitivity to both cues, then things get more complicated.

There is mounting evidence that children sometimes demonstrate sensitivity to cues when multiple cues act together but not when each cue is presented separately (e.g. Bittner & Kuehnast, 2012; Song & Fisher, 2005, 2007). Thus, we might see that, unlike adults, young children have more of a tendency toward first-mentioned responses when the gesture is consistent with the order-of-mention cue than when there is no gesture, but no influence of gesture when it goes against order-of-mention (and no evidence of a first-mentioned bias in the absence of gesture), results that would demonstrate weak but real influences of both cues. Sensitivity to both cues might alternatively manifest as confusion when the two cues conflict, as the children struggle with prioritizing the more informative pragmatic cue (gesture, as adults do) over one derived from a statistical pattern in the input (order-of-mention).

There are therefore several different questions addressed in our analyses. We first examine children's responses in the absence of gestural cues to interpretation, looking to see if there is any evidence for a first-mentioned bias. This provides a baseline against which we can assess the effect of gestures. We then examine whether first-mentioned interpretations increase with the presence of gestures consistent with a first-mentioned interpretation, and explore whether this pattern changes with age. Finally, we examine the effect of gestures that are inconsistent with a first-mentioned interpretation, asking whether first-mentioned responses decrease, or if there any evidence for greater confusion when the cues conflict.

## METHOD

### Participants

A total of 120 four-, five-, seven-, and eight-year-old children participated. They were divided into younger children (4–5) and older children (7–8), with 20 children per condition per age group, and an approximately equal number of boys and girls. These ages were selected based on research suggesting that during this period children begin to show sensitivity to (younger children) and eventually master (older children) various cues for ambiguous pronoun resolution, such as gender and order-of-mention. Thus, we might expect gesture to be especially helpful to the younger children when the sentences lack a constellation of cues (which can lead to cue use earlier than this; see Song & Fisher, 2005, 2007), and might be used in more adult-like ways by the older ages.

Child participants were recruited from and tested at childcare and after-school programs in the Berkeley area, a local science museum, and in the lab. They received stickers or small toys for their participation (unless they were in a school that did not allow it). All participants were native English speakers with no history of sign language experience.

### Procedure

Participants were seated in front of a video monitor (in the lab) or laptop computer with external speakers (outside the lab) on which they watched eight video clips of a narrator (the first author) telling short stories. The narrator was shown from the waist up, and her hands and face were clearly visible. (The narrations were identical to those previously used with adults in Goodrich Smith & Hudson Kam, 2012, with the exception of one in which the names of the characters were changed to ones the younger children were more familiar with.) Five of the eight vignettes were experimental items, each containing an ambiguous pronoun. (We used a relatively small number of items to ensure that the youngest children could complete the entire study, and within the limited time frame allowed by at least one setting in which we collected data.) These stories contained two characters of the same gender. The characters were first introduced and then mentioned again by name before the narrator described an action performed by only one of the characters using a pronoun. While the order of the characters was not counterbalanced, we made an effort to ensure that both characters were equally likely to be the antecedent. For instance, we specifically avoided using one character who can't speak paired with one who can, if the story involved talking. The two characters were introduced in such a way as to avoid the influence of syntactic constituency as much as possible; the case of the ambiguous pronoun either matched the case used for both participants or neither participant. (The latter was true of one item, in which the ambiguous pronoun was a possessive.) A sample ambiguous narration is shown in Table 1. (The complete list is presented in the ‘Appendix’.)

**Table 1.** Sample stimuli

Narrative type	Narrative
Ambiguous pronoun narrative	Annie and Sarah are having a picnic in the park. They have a lot of food with them. Annie is carrying the picnic basket, and Sarah has a blanket to sit on. She's excited about the cookies.
Filler narrative	Sally and Tim are swimming in a pool. Sally likes to jump off the diving board. Tim doesn't like it when Sally jumps, because she splashes water.

Participants also heard three filler stories with two characters that did not contain ambiguous pronouns. Pilot testing revealed that children almost always answered the filler story questions correctly; thus they were used as the first, middle, and last narrations to allow the child to begin and end the study on a positive note. A sample filler narrative can be seen in Table 1. Each narration was approximately 12·5 seconds long.

Following each narration, the image on the screen paused and the experimenter asked the participant a question. The questions were asked live by the experimenter rather than a recorded voice-over in an effort to maintain child interest in the task, as well as to limit the time gap between the narration and the question. For the experimental items, the question asked about the identity of the referent of the ambiguous pronoun. For instance, the question for the ambiguous pronoun narrative presented in Table 1 was “Who is excited about the cookies?” For the filler narratives, participants were asked about other details from the story. For example, after the filler narration presented in Table 1, children were asked “Does Tim like it when Sally jumps?”

Participants were instructed to listen carefully to each story and answer each question out loud as it was asked. The experimenter wrote down each child's response, and a digital recorder, placed on a table next to where they were seated, was also used to record participants' responses.

### Experimental manipulation

**Fig. 1.**
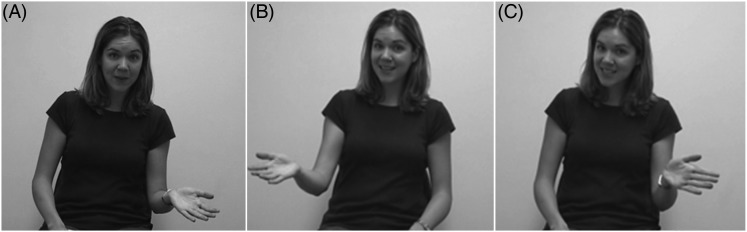
Example of (A) Localizing Gesture with first-mentioned name, (B) Localizing Gesture with second-mentioned name, and (C) Co-referential Gesture consistent with order of mention with pronoun. Note that the gesture in C is on the same side as that in A, but not in exactly the same location.

Participants were assigned to one of three conditions. The script of the narrations was identical for all conditions (i.e. all participants heard the same narratives and questions). What varied was the presence/location of gestures that co-occurred with the ambiguous pronoun. The initial localizing gestures associated with the characters' names located each character in abstract gesture space in front of the narrator. The co-referential gesture that occurred with the pronoun had the same general form as the first two gestures, and was produced on the same side as one of the previous gestures. It is co-referential by virtue of the fact that it goes back to a space previously associated with one of the characters. In the Order-of-Mention (OoM) condition the narrator (WGS) gestured while saying both names, and when saying the pronoun gestured in the location that matched the first-mentioned name. An example of this is shown in Figure 1. In the Against-Order-of-Mention (AOoM) condition the narrator gestured toward the location that went with the second-mentioned name when producing the ambiguous pronoun. In the final condition, No Gesture (NG), the narrator kept her hands in her lap while speaking. This condition was designed as a baseline measure of how children interpret the pronoun without the presence of gesture. Interpretation in this condition served as the metric against which interpretation in other conditions, where gesture served as an additional cue, was compared.

The gestures themselves involved an open palm handshape. Although, to our knowledge, all of the extant research on gesture interpretation in children has used either iconic gestures or points, there is no a priori reason to believe that the gestures in our stimuli would cause problems for the children. First, they were based on the gestures that the narrator produced naturalistically in conjunction with the stories. Thus, they were designed to appear as naturalistic as possible. Second, adults in our previous study (Goodrich Smith & Hudson Kam, 2012) utilized them to interpret the pronouns even in the very first vignette they saw (i.e. they did not have to learn how to interpret them). Moreover, adult participants generally did not report noticing the gestures in the videos, despite the fact that their interpretations were affected by the gestures, suggesting that they were perceived as quite natural. This accords with work examining gesture and co-reference. While iconic gestures are also quite natural in this situation, research shows that speakers often use quite neutral gestures (including a stationary open handshape) during anaphoric reference to people (Foraker, 2011). Moreover, given the nature of the narratives, the only iconic representations that would make sense would be iconic representations of aspects of the verb in the sentence with the pronoun. If the gestures had encoded aspects of the verb in the sentence in which the character had been introduced, they would have much more explicitly included disambiguating information. This in turn would have removed the co-referential aspect of interpretation. That is, the children would not have to encode and remember the initial locations associated with each character and associate the pronoun-gesture with one of those locations in order to interpret the pronoun if they could rely on iconic gestures representing aspects of the character itself. This is an essential feature of pronominal reference, the co-referential aspect of meaning assignment, and so something we intentionally wanted in the gestures. (Note that the gestures sometimes did include non-disambiguating iconicity, such as movement associated with the verb produced in the sentence with the pronoun.) For the same reasons we did not use points: points to abstract locations previously associated with the characters do not occur in natural speech (Foraker, 2011), and points to things or pictures present in the environment would circumvent having to do co-reference in order to interpret the pronoun.

### Coding and analysis

Participants’ responses were coded as selecting the first-mentioned character, the second-mentioned character, or other (e.g. when asked “Who had an umbrella?” replying “They both did” or “I don't know”) as the referent for the ambiguous pronoun. Situations where the child needed to be prompted, a possible index of difficulty in interpreting the pronoun, were also noted. (In these instances, the response after being prompted, if one was given, was recorded as the interpretation.)

## RESULTS

Before examining first-mentioned character responses, we first pulled out the relatively small number of responses that were coded ‘other’. This consisted primarily of “I don't know” responses or “both of them”. Note that if the child responded with a single name after being prompted following an “I don't know” response, that response would be coded according to the name eventually provided, not the initial “I don't know”. Thus, the unresolved ‘other’ responses made up a subset of the data that was later removed from further analysis. ‘Other’ responses were relatively rare: only 20 children out of 120 had any such responses, and only two had more than one, making an analysis difficult. They were mostly younger children (17/20), but not all, and they were in all three conditions, although they were numerically less frequent in the OoM condition (3/20, all in the younger age group), where the two cues point to the same interpretation. We had hypothesized that these responses might be more frequent in children in the AOoM condition where the two cues clash, but this was not the case. Due to the rarity of ‘other’ responses, they are not included in the analysis below.

### Baseline performance

**Fig. 2.**
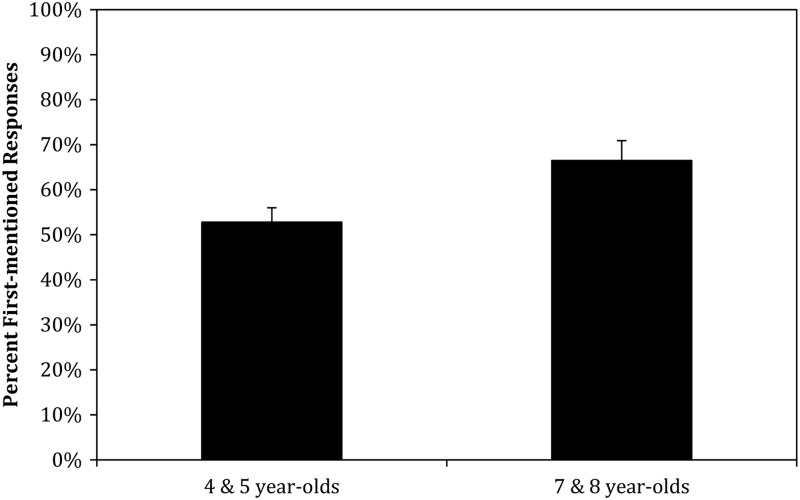
Mean percent first-mentioned responses by age group in the NG condition.

Recall that the NG condition, with no cues to pronoun reference except order-of-mention, allows us to see what participants’ responses are like in the absence of any gestural cue to pronoun interpretation. This condition therefore serves as our baseline condition. It also allows us to explore the development of the order-of-mention tendency in children when other cues to pronoun interpretation such as topic and grammatical role are held constant. Figure 2 displays the percent of first-mentioned responses for the NG condition by age group. These percentages (and all others that follow unless specified) were calculated over all specific responses, rather than all responses. That is, any responses that fell into the ‘other’ category were not included in the denominator.

Although Figure 2 shows that the older children are more likely to interpret the pronoun as referring to the first-mentioned character, an ANOVA^1^ shows no significant effect of age (*F*(1,38) = 1·642, *p* = ·208, *η*_*p*_^2^ = ·041), so we compared the children's performance to chance (50%) for the data set as a whole, i.e. with the data from both ages combined, to establish whether the responses showed a first-mentioned bias. (Note that, unless otherwise specified, all comparisons are 2-tailed.) The results show that the children do show a first-mentioned bias (*t*(39) = 2·119, *p* = ·041, Cohen's *d* = ·335).

Despite the non-significant effect of age in the ANOVA, an inspection of the figure shows a substantial difference between the two age groups; the younger group hover around 50% first-mentioned responses whereas the mean of the older children is quite a bit above. This pattern, data which appear to be quite different from different groups in the absence of a significant difference, can result from a high degree of variation either in one or both groups. We conducted some exploratory *t*-tests comparing each age group to chance individually. These analyses show that only the older children as a group select the first-mentioned character more often than chance (4–5-year-olds: *t*(19) = 0·182, *p* = ·857, Cohen's *d* = ·041; 7–8-year-olds: *t*(19) = 3·789, *p* = ·001, Cohen's *d* = ·846). Notably, the 95% CI for the difference between the mean and chance for the seven- to eight-year-old children (0.0739 to 0.2561) is entirely positive – it does not include values with no difference or a negative difference (the latter of which would indicate a preference for the second-mentioned character) – unlike the CI for the four- to five-year-olds (–0·1309 to 0·1559), which dips substantially into negative difference territory. It is also much tighter with a range of 0·1822 in contrast to the CI for the younger children (0·2868), which includes a much larger range of values.

Taken together, these results suggest that children at the younger ages do not yet have a preference for the first-mentioned name when order-of-mention is the only cue available, but that by age seven children are beginning to show the adult-like order-of-mention tendency as described in Goodrich Smith and Hudson Kam (2012) for this stimuli. In that study we found that adults in the No Gesture condition interpreted the pronoun as referring to the first-mentioned character 68% of the time, which is very similar to the seven- and eight-year-olds in the present study (66·5%). If we only consider the first- and second-mentioned character responses, as with the children, then the adult percentage of first-mentioned character interpretations rises to 81%. However post-hoc analysis using a 2-sided Dunnett test shows that even the higher adult percentage is not statistically different than that of the older children (*p* = ·155; 95% CI of the difference between the two age groups (using transformed scores) = –3·411 to 0·4718), although it is different than the younger children (*p* = ·014, 95% CI = –4·333 to –0·449918).

### Gesture cue

**Fig. 3.**
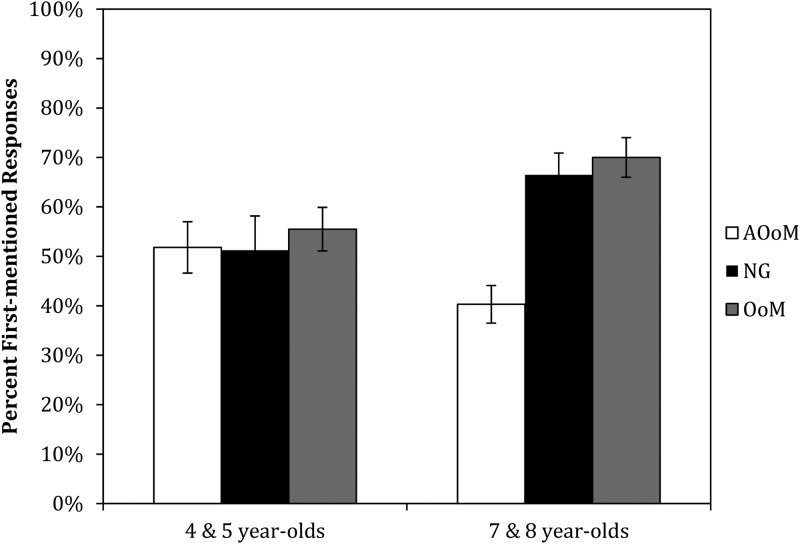
Mean percent first-mentioned responses by age group and condition (AOoM = Against Order of Mention, NG = No Gesture, OoM = (consistent with) Order of Mention).

Figure 3 shows the percentage of the time that participants interpreted the ambiguous pronoun as referring to the first-mentioned character, by age group and condition. In an overall ANOVA nothing reached the ·05 criterion for significance (age group: *F*(1,114) = 3·615, *p* = ·06, *η*_*p*_^2^ = ·031; condition: *F*(2,114) = 2·815, *p* = ·064, *η*_*p*_^2^ = ·047; interaction: *F*(2,114) = 1·1935, *p* = ·149, *η*_*p*_^2^ = ·033).

The non-significant effects of age group and condition are not surprising, as the older children are not consistently more likely to interpret the ambiguous pronoun as referring to the first-mentioned character and no effects of condition are apparent in the younger children; therefore, there are unlikely to be any consistent effects of condition. It does appear from the figure that there is an interaction; however, this is not significant either. This is likely due to the fact that there is only one difference between the two age groups that is not consistent with an overall higher rate of first-mentioned responses in the older children, namely, the lower rate of first-mentioned responses in the second-mentioned condition. This seemingly small difference between the patterns is not enough for the interaction between the two factors to be significant. However, this one difference makes the older children's responses mirror the adults’ – no effect of gestures consistent with order-of-mention, but an effect of gestures that go against the order-of-mention pattern.

Given that the older, but not younger, children's responses appear to show the same pattern as the adult data in our previous study, we performed further analyses examining the influence of condition for each age group separately, despite the non-significant interaction. Condition was not significant for the younger age group (*F*(2,57) = 0·027, *p* = ·973, *η*_*p*_^2^ = ·001), but it was for the seven- and eight-year-olds (*F*(2,57) = 10·552, *p* < ·001, *η*_*p*_^2^ = ·270). To further explore this result, we compared performance in the OoM and AOoM conditions to performance in the baseline NG condition for the older children using a 2-sided Dunnett test. First-mentioned responses were significantly less frequent in the AOoM condition than the NG condition (*p* = ·002), but there was no difference between the OoM and NG conditions (*p* = ·509). Moreover, the 95% CIs of the difference between the two means (mean proportion of first-mentioned responses in the NG condition – mean proportion of first-mentioned responses in the OoM condition, likewise for NG and AOoM) include both positive and negative values for the comparison between the NG and OoM conditions (–0·4882 to 1·2517, computed using transformed scores), but the CI for the comparison between the NG and AOoM conditions contains only negative values (–2·1689 to –0·4289).

Thus, at the younger ages, the gestures do not appear to be having any effect on pronoun interpretation, while at the older ages the gestures decrease the likelihood of a first-mentioned character interpretation when the gesture conflicts with that interpretation, but do not significantly increase the likelihood of a first-mentioned character interpretation when the gesture accords with that interpretation. Given the coding scheme used here, we can also say on the basis of these data that for the older children, seeing co-referential gestures that indicated the second-mentioned character was the intended referent made them more likely to interpret the pronoun as referring to the second-mentioned character. This is the same pattern we saw with adult participants in our previous study – co-referential gestures consistent with the first-mentioned character increased slightly but not significantly, while gestures consistent with the second-mentioned character led to increased second-mentioned character interpretations, as compared to participants who saw no gestures.

**Fig. 4.**
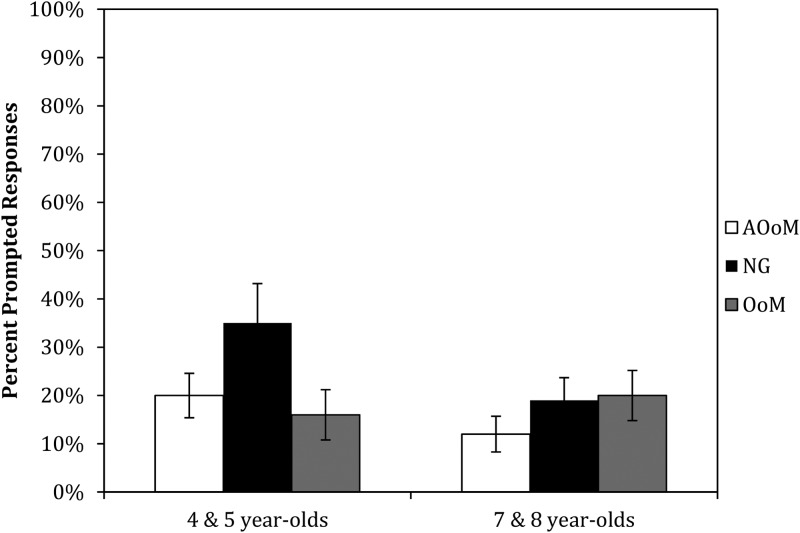
Mean percent of responses that were prompted by age group and condition.

But children in the AOoM condition did not interpret the pronoun as referring to the second-mentioned character all the time, despite the very clear information present in the gesture that indicated the speaker's intended referent. Although this same pattern was seen in the adult participants in Goodrich Smith and Hudson Kam (2012) – a significant but not complete shift in interpretation when the gestures went against the order-of-mention tendency – it is possible that the presence of two conflicting cues simply confused the children. To assess whether children were more confused when gesture conflicted with order-of-mention, we examined how often children required prompting, that is, we computed the number of times children did not answer the initial question, and thus required prompting (for example, when the experimenter asked “Do you remember if it was Annie or Sarah?”). Figure 4 shows the percentage of total responses that required prompting, by condition for the two age groups. (Note that these are computed over all responses, not just ones that were resolved to the first- or second-mentioned character.) An examination of the figure shows that prompts were most common for the younger children in the absence of any gestures, which might suggest that the gestures do help the youngest children interpret the pronouns more easily. However, the statistical analysis revealed no significant effects (age group: *F*(1,114) = 1·583, *p* = ·221, *η*_*p*_^2^ ^=^ ·014; condition: *F*(2,114) = 1·143, *p* = ·323, *η*_*p*_^2^ = ·02; age group × condition: *F*(2,114) = 1·361, *p* = ·261, *η*_*p*_^2^ = ·023). This statistical result is not surprising given the number of children who had no prompted responses (55/120), and the fact that of those children who were prompted, most were only prompted once or twice (51/65).

## DISCUSSION AND CONCLUSION

The aim of the current study was to investigate whether children can rely on information present in co-speech gestures to help resolve ambiguities in speech. We were specifically interested in whether children make use of a gestural cue to pronominal reference. Thus, an important first step was to establish how children interpreted the stimuli in the absence of any co-referential gestures. We therefore first established the degree to which children aged four to eight showed a first-mentioned bias for these stimuli which contained no other cues to pronoun interpretation. This allowed us to assess the age at which children use the order-of-mention cue by itself (i.e. when it is the only cue present in the speech), something that had not been established in previous studies. The overall analysis showed a first-mentioned bias for all of the children. However, in the more exploratory analysis where we broke things down by age, it became apparent that this was really being driven by the oldest children. In our youngest age group (4- and 5-year-olds) we found no evidence for an adult-like first-mention bias. Children in the older age group, in contrast, look very much like the adults in Goodrich Smith and Hudson Kam (2012).

To assess the impact of the gestures on interpretation we asked several questions. First, whether first-mentioned interpretations increased when the children saw gestures that were consistent with the first-mentioned bias. Although first-mentioned responses were numerically more frequent in both age groups in the OoM conditions as compared to the NG conditions, these differences were slight and non-significant. Thus, as with adults, the presence of gestures that accord with the first-mention bias does not seem to affect interpretations much in children over this age range.

We did, however, find that first-mentioned responses go down (and second-mentioned responses go up) when children saw gestures that indicated that the second-mentioned character was the intended referent of the pronoun, at least for the older children. Thus, the older children do show sensitivity to the gestures.

Additionally, we examined a potential measure of confusion that would indicate some sensitivity to the cues, namely, the incidence of prompting, but we found little evidence that more prompting was required when the two cues were in conflict (in the AOoM condition). It was the case that younger children in the NG condition required more prompting than those in the two gesture conditions. However, given that this was true whether the gestures were in accord with or went against the order-of-mention tendency, this seems to be a chance finding (especially given the fact that nothing was significant in that analysis). ‘Other’ responses are another possible sign of confusion, however, they were too rare to analyze statistically; indeed, they were rare enough that we removed them from most of our analyses. However, they showed an interesting trend. They were less frequent in the OoM condition than the two gesture conditions, even for the youngest children. This might suggest that the convergence of cues in that condition did lead to better performance, which is consistent with other studies finding an impact of cues to pronoun interpretation in even younger children when there are several cues and they converge (Song & Fisher, 2005, 2007). However, given that this reduction in other responses was not accompanied by an increase in first-mentioned responses, we do not wish to make too much of this pattern.

On the metrics just discussed, then, it appears that only the older children show any real sensitivity to either the first-mentioned tendency or gesture. The first result is in accord with the data in Arnold *et al.* (2007a), but not other studies which have found earlier sensitivity to order-of-mention (Pyykkönen *et al.*, 2010; Song & Fisher, 2005, 2007). However, recall that previous studies examining order-of-mention in children have always also included other cues, such as grammatical role or topic (sometimes both), and there is some evidence that multiple cues which all serve to highlight the same antecedent are required to show evidence of cue use in young children. Indeed, when discussing possible outcomes we raised the possibility that we might only see an effect of gesture when it accorded with order-of-mention and vice versa (we might only see evidence for an order-of-mention effect in the OoM condition) based on such evidence. However, this was not the case. The two cues did not seem to reinforce each other in either age group: the younger children were not sensitive to either, alone or in concert, and the older children were affected by both the order-of-mention cue and the order-of-mention incongruent gestures, but order-of-mention congruent gestures did not significantly increase the first-mentioned interpretations. The fact that the younger children failed to show a first-mentioned response bias even in the condition with multiple cues strongly suggests that order-of-mention in and of itself is not a pattern that the younger children have picked up on yet, and further suggests that the studies finding evidence for a first-mentioned bias in younger children are really finding a bias for interpreting subjects as antecedents.

As pointed out by Hartshorne *et al.* (2011), the studies that have first-mentioned bias in children all used eye-tracking as a measure, in contrast to the present study that only used explicit responses to questions. It is not unusual to find a weak sensitivity to something displayed in eye-movements that is not apparent in explicit responses (e.g. Beyer & Hudson Kam, 2009), so it is possible that the difference between our results (with respect to the first-mentioned bias) are methodological. However, Hartshorne *et al.* (2011) found evidence for the bias in both eye-movements and explicit responses, while Arnold *et al.* (2007a) found it in neither. Note that Arnold *et al.,* looked at a different time delay from the stimulus as studies who found an effect, a fact pointed out by Hartshorne *et al.* (2011). They also had fewer redundancies in their stimuli than most of the other studies, suggesting that younger children will show more adult-like interpretations given highly favorable conditions. Thus, we think it more likely that our lack of a first-mentioned bias in the younger children is due to the fact that order-of-mention truly was the only non-gestural information the children had, something that is generally not the case, either in experiments, or in natural speech input.

One might ask: Why not use other cues such as grammatical concordance or stress instead of order-of-mention in the no-gesture condition to establish a baseline? Our choice of order-of-mention was two-fold. First, order cannot be avoided, when there are multiple names mentioned, one is always going to have to be first. Second, we expected it to have a weak effect on interpretation. Our intention was not really to put two cues in conflict and see which one ‘won’ or had the stronger effect on interpretation, it was to see if children could use the information potentially available to them in gesture. True conflict between cues to reference, e.g. between gesture and stress, is almost certainly rare in the speech children hear and would likely just serve to confuse them; adult speakers know what they are talking about, and while they often provide different information in speech and gesture, this information is typically additive, not conflicting (McNeill, 1992; cf. Alibali & Goldin-Meadow, 1993, for evidence that young children will produce gesture–speech mismatches when in a transitional state of knowledge). Thus, while our study can speak to the development in the use of order-of-mention information by itself as a cue, this is a secondary, rather accidental, aspect of our study.

With respect to the primary question this research was designed to address, the impact of gesture on interpretation, it is rather surprising (to us at least) that the children in the current study did not utilize gesture until relatively late, given the previous research demonstrating that much younger children are capable of perceiving information conveyed through gesture (e.g. Goodrich & Hudson Kam, 2009; Kelly, 2001; Morford & Goldin-Meadow, 1992). Two possibilities for this discrepancy are rather easily dismissed. The first is that children at the younger ages in our study are simply unable to associate space with a referent. This seems unlikely to be the correct explanation, given work on children learning signed languages (where such mappings are often part of the grammar of the language), who make such mappings in comprehension, if not production, well before the age of six (see Schick, 2003, for a summary). The second is that pronoun interpretation is a closed process sensitive only to the accessibility of the possible referents. On this explanation, the gestures investigated in our study simply cannot affect interpretation since they do not directly affect accessibility or salience in a mental model. However, that would suggest that gestures should never affect interpretation, something not true of adults (Goodrich Smith & Hudson Kam, 2012; Nappa & Arnold, 2014), or the older children in this study.

Other possibilities are not easily adjudicated by our data, nor other extant work. One possibility is the nature of the gesture–meaning mapping in our study. Previous studies demonstrating younger children's ability to interpret gesture used gestures that mapped onto their meanings in much more obvious ways, direct points at an object, for instance, or movements mimicking an action performed by a toy, requiring very little interpretation on the part of the child. Co-referential localizing gestures, in contrast, require the child to associate the space in which a gesture occurred with the character mentioned while the gesture was produced, and then later remember which spaces were associated with which characters. This may place too heavy a cognitive load on young children. As discussed previously, this aspect of the gestures was intentional, as it mimics co-reference in speech – referents are introduced and then referred back to, and it would be true of any gestures of this type occurring in natural, spontaneous speech. Thus, it is not a feature of gesture interpretation we would wish to remove. It is entirely possible (indeed, quite likely) that children in our youngest age group could use points directed toward people or objects to interpret a pronoun. But that would not be co-reference, and so does not answer the question we set out address. Another possibility is that the open-hand-style deictic gestures were unfamiliar to the children, leading to poor performance. If so, then they might do better with points, a gesture form they are known to integrate with speech much earlier than this (e.g. Morford & Golden-Meadow, 1992). (Although it is not entirely clear what might change in children's experiences between 5 and 7 with open-hand deictics such that they would suddenly succeed at 7.) One way to assess this would be to run a similar study using points instead of open-hand gestures. Points seem very unnatural given the context, however, at least for North Americans. Although people do point to locations in space abstractly associated with real-world entities – for instance, when trying to remember the name of someone who had been seated in a nearby location, a person might point to the location, almost in an effort to retrieve the name – these points are again not co-referential with a previously established referent. (Moreover, they tend to occur with much less fluency in speech, and seem less well integrated with speech. Indeed, they seem to stand in for speech instead of being complementary to it.)

Alternatively, the lack of use of the gestures by the younger children may have more to do with the aspect of language represented than any features of the gestures themselves. Children may notice iconic gestures representing verbs, for instance, earlier than the much more abstract co-referential gestures precisely because anaphora is acquired comparatively late as compared to verbs. That is, if gesture and speech truly form an integrated system in terms of comprehension as well as production (an idea supported by a wide body of research; e.g. McNeill, 1992, 2005; Özyürek & Kelly, 2007), and children are at an age when they are not yet linguistically ready to understand the co-referential relations, as at least some previous research suggests (Karmiloff-Smith, 1986), then the gestures will not be interpretable as such.

Another possibility is that, like speech-internal cues, listeners must learn to use gesture cues, and that a certain amount of exposure is needed before a cue will be used to guide interpretation. Although co-referential localizing gestures do occur in natural speech contexts, they do not occur as frequently as speech-internal cues, which by definition occur in every sentence (although they may not be the same in every sentence, i.e. it is not the case that in every sentence co-reference lines up with the speech-internal cues discussed in the ‘Introduction’). It may therefore be the case that the younger children in our study simply did not yet have enough exposure to these types of gestures to consistently use them in their interpretations.

Although our intention was not to examine the development of the first-mention bias in and of itself, our results do contribute something to the debate about whether or not it is a factor in pronoun interpretation (see, e.g. Kehler, 2002). On our reading of the literature, surface factors like grammatical congruence and order-of-mention are thought to increase the accessibility of an entity in the listener's mental representation, thereby increasing the likelihood of it being selected as the antecedent for a pronoun, all else being equal. Why certain things make an entity more accessible is open for discussion, but it is plausible that, for at least some cues, speakers become sensitive to the patterns present in the input via some sort of associative learning, something we know humans are very good at (see, e.g. Saffran, Aslin & Newport, 1996). Pronouns do often refer to first-mentioned entities; thus, it is a pattern available to be learned. While it may not be a strong effect in everyday pronoun interpretation for adults or children due to the availability and strength of other factors in most natural language productions, our data suggest that it is eventually learned, such that it can affect interpretation in the absence of other cues. However, our data also raise the possibility that studies reporting earlier evidence of sensitivity to order-of-mention might actually be demonstrating sensitivity to the other cues, i.e. grammatical information or topic (which seems to be very important for young children; Spenader, Smits & Hendriks, 2009), not order-of-mention.

In summary, the current study joins previous work exploring children's ability to rely on various discourse cues to inform pronoun interpretation. It expands on our previous research with adults (Goodrich Smith & Hudson Kam, 2012) and suggests that gesture is one of many cues that children can rely on to interpret the meaning of ambiguous pronouns, at least when they are older. More broadly, our results contribute to a growing body of research (e.g. Broaders & Goldin-Meadow, 2010; Goodrich & Hudson Kam, 2009; Stanfield *et al.*, 2014) suggesting that, like adults, children are sensitive to information contained uniquely in gesture. However, our results also suggest that one can't simply assume that gesture will be used by children in every potentially relevant situation. Instead, sensitivity to gestures might take time (and possibly experience) to develop.

## Footnotes

1 We performed a log-odds transformation on the data before all ANOVAs and *t*-tests that compared one group's data to another's (but not when we compared performance to chance). Zeros were changed to ·001 and 1 s to ·99 prior to transformation.

## Appendix

Ambiguous pronoun narrations (some based on those in Arnold *et al.*, 2007a)

**Table:**

Narration	Question
C1^*^ and C2 are good friends. C1 is walking up a hill and C2 is at the top. He has an umbrella, which is good, because it looks like it's going to rain.	Who has an umbrella?
C1 and C2 were walking towards each other on the street. When they saw each other, C1 smiled, and C2 grinned. He said “It's good to see you!”	Who said “It's good to see you”?
C1 and C2 are having a picnic in the park. They have a lot of food with them. C1 is carrying the picnic basket, and C2 has a blanket for them to sit on. She's excited about the cookies.	Who is excited about the cookies?
C1 and C2 are good friends, but they live very far apart, and hardly ever get to see each other. Recently, C1 visited C2 during his summer vacation. They were very happy to see each other.	Who had a summer vacation?
My cat's name is C1. C1 has brown fur. My neighbor's cat is named C2. C2 has white fur. C1 and C2 don't like each other. He's afraid of him.	Who is afraid of the other cat?

notes: ^*^ Names have been replaced with C1 for the first-mentioned character and C2 for the second-mentioned character for reasons of trademark. The fully specified stimuli are available by request from the authors.
